# Harnessing Insect Chemosensory and Mechanosensory Receptors Involved in Feeding for Precision Pest Management

**DOI:** 10.3390/life15010110

**Published:** 2025-01-16

**Authors:** Tingwei Mi, Chengwang Sheng, Cassidy Kylene Lee, Peter Nguyen, Yali V. Zhang

**Affiliations:** 1 Monell Chemical Senses Center, Philadelphia, PA 19104, USA; tmi@monell.org (T.M.); csheng@monell.org (C.S.); cassidylee@monell.org (C.K.L.); 2 Department of Pesticide Science, Anhui Agricultural University, Hefei 230036, China; 3 Department of Biology, University of Pennsylvania, Philadelphia, PA 19104, USA; petervn@sas.upenn.edu; 4 Department of Physiology, The Institute for Diabetes, Obesity, and Metabolism, Perelman School of Medicine, University of Pennsylvania, Philadelphia, PA 19104, USA

**Keywords:** insect, chemoreceptor, mechanoreceptor, ion channel, feeding, reproduction, pest management

## Abstract

Chemosensation and mechanosensation are vital to insects’ survival and behavior, shaping critical physiological processes such as feeding, metabolism, mating, and reproduction. During feeding, insects rely on diverse chemosensory and mechanosensory receptors to distinguish between nutritious and harmful substances, enabling them to select suitable food sources while avoiding toxins. These receptors are distributed across various body parts, allowing insects to detect environmental cues about food quality and adjust their behaviors accordingly. A deeper understanding of insect sensory physiology, especially during feeding, not only enhances our knowledge of insect biology but also offers significant opportunities for practical applications. This review highlights recent advancements in research on feeding-related sensory receptors, covering a wide range of insect species, from the model organism *Drosophila melanogaster* to agricultural and human pests. Additionally, this review examines the potential of targeting insect sensory receptors for precision pest control. Disrupting behaviors such as feeding and reproduction emerges as a promising strategy for pest management. By interfering with these essential behaviors, we can effectively control pest populations while minimizing environmental impacts and promoting ecological balance.

## 1. Introduction

**Figure 1:**
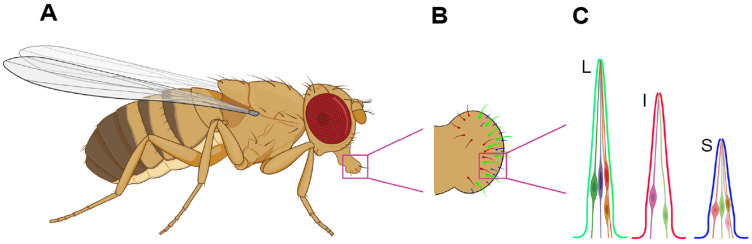
**Taste organs and gustatory receptor neurons (GRNs) in the fruit fly, *Drosophila melanogaster*.** (**A**) The fly’s proboscis serves as a primary taste organ. (**B**) The fly’s labellum contains L- (green), I- (red), and S- (blue) type sensilla. (**C**) Each L- or S-type sensillum typically houses four GRNs, whereas each I-type sensillum contains two GRNs.

In insects, chemosensation (taste and smell) and mechanosensation play essential roles in various physiological processes, including feeding [1], nutrient metabolism [2], and reproduction [3,4]. Among these senses, taste is particularly important for feeding behavior as it enables insects to identify beneficial nutrients while avoiding harmful substances. Through a diverse array of taste receptors [1,5,6], insects can detect the presence of sugars [7,8,9], amino acids [10,11,12], fatty acids [13,14,15,16,17], low levels of salts [18,19,20] and acids [21,22,23,24], and other essential nutrients. Taste also acts as a defense mechanism, helping insects detect and avoid unpalatable or toxic substances [25,26,27], such as bitter compounds, high concentrations of salts [18,28,29,30] and acids [21,22,31], and alkalis [32,33]. Among insect species, the fruit fly, *Drosophila melanogaster* (Figure 1A), is widely used as a model for studying taste perception due to its well-understood genome and genetic tools [5,6,34]. On the surface of the labellum of the fly’s proboscis (mouthpart) (Figure 1B) are hair-like structures called taste sensilla, which have open ends that allow them to sample food from the environment [35,36]. Each half of the fly labellum contains 31 sensilla, categorized into small (S), intermediate (I), and large (L) types [5,36]. These sensilla house gustatory receptor neurons (GRNs) (Figure 1C), which convert chemical signals into neuronal responses, ultimately guiding feeding behavior. Distinct GRNs express specific taste receptors that detect a variety of tastants, such as sugars, bitter compounds, acids, and salts. While *Drosophila melanogaster* is an excellent model for studying sensory biology, there is considerable diversity in the anatomy of sensory organs and receptor repertoires across insect species. Therefore, findings from *Drosophila melanogaster* may not be directly applicable to all insects. Nonetheless, the insights gained from *Drosophila melanogaster* provide a valuable foundation for advancing our understanding of chemosensation and mechanosensation in other insect species.

Here, we provide an overview of the diverse chemosensory and mechanosensory receptors in insects involved in host-seeking and feeding, highlighting their physiological roles and ecological importance. These receptors allow insects to detect and interpret environmental cues related to food sources, enabling them to make decisions that enhance feeding efficiency, reproductive success, and overall fitness [1,3]. Importantly, these sensory receptors represent promising targets for the development of innovative pest control strategies [37,38,39]. Genetic approaches could be employed to impair insects’ ability to detect critical cues like food, thereby reducing their survival and reproduction [40]. Chemical strategies could involve designing compounds that block or overstimulate these receptors, disrupting feeding and mating behaviors [41]. Such targeted methods offer environmentally friendly, species-specific alternatives to broad-spectrum insecticides, potentially providing a sustainable solution for pest management.

## 2. Gustatory Receptors (GRs)

In insects, many taste receptors belong to the GR superfamily [42,43], which shows remarkable diversification across taxa [5]. In *Drosophila melanogaster*, its GR family comprises about 68 GR members, each involved in detecting a wide range of tastants from sugars to bitter compounds [43,44,45]. In mosquitoes, such as the African malaria vector *Anopheles gambiae* and the yellow fever vector *Aedes aegypti*, there are 76 and 91 GR members, respectively [46,47], some of which help detect carbon dioxide emitted by hosts [48]. In Lepidopterans, including *Bombyx mori* [49], *Heliconius melpomene* [50], *Helicoverpa armigera*, *Spodoptera frugiperda*, and *Spodoptera litura* [51], their GR family has expanded significantly. Specifically, the polyphagous pests *Helicoverpa armigera*, *Spodoptera frugiperda*, and *Spodoptera litura* show large expansions of the GR family, with 197, 232, and 237 members, respectively, likely due to their need to adapt to a wide variety of host plants [51].

**Figure 2:**
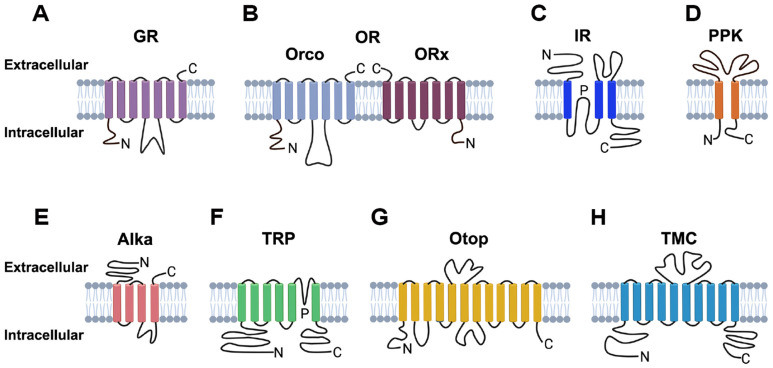
**Transmembrane topologies of different families of chemosensory and mechanosensory receptors in the fruit fly, *Drosophila melanogaster*.** (**A**) Gustatory receptor (GR); (**B**) Olfactory receptor (OR) complex, consisting of an ORx and the coreceptor Orco; (**C**) Ionotropic receptor (IR); (**D**) Pickpocket (PPK); (**E**) Alkaliphile (Alka); (**F**) Transient receptor potential (TRP); (**G**) Otopetrin (Otop); and (**H**) Transmembrane channel-like (TMC). **N** represents the amino terminus, **C** represents the carboxyl terminus, and **P** indicates the pore loop.

In contrast to mammalian taste receptors, which are primarily G protein-coupled receptors (GPCRs), insect GRs exhibit significant differences in both protein sequence and transmembrane topology (Figure 2A) [52]. Recent advancements, particularly through single-particle cryo-electron microscopy (Cryo-EM), have shed light on the structural organization of certain insect GRs. For instance, the fly taste receptor DmGr43a has been shown to assemble into tetrameric ion channel complexes [53]. Likewise, in the silkworm, *Bombyx mori*, the fructose taste receptor BmGr9 forms a tetrameric cation channel that is directly gated by fructose [54,55,56]. These structural studies imply that insect GRs may function more broadly as ion channels rather than as GPCRs, indicating a fundamental difference in how taste is transduced in these organisms.

Among the identified bitter GRs in fruit flies, certain members, including DmGr33a, DmGr39a, DmGr66a, and DmGr89a, act as core receptors implicated in detecting various aversive chemicals [44,57], while others, like DmGr8a, are finely tuned to specific bitter compounds [26]. The process of detecting bitter compounds involves intricate interactions among multiple GR subunits, underscoring the complexity and depth of bitter taste perception. Similarly, sweet taste receptors responsible for sensing sugars also belong to the GR superfamily. Notable examples include DmGr5a, required for sensing trehalose [7,58]; DmGr43a for fructose; DmGr64a for sucrose and maltose; and DmGr64f, broadly tuned to a wide array of sugars except for fructose [59]. Sweet GRs are proposed to be activated in different ways: some, like DmGr43a, may function alone as homomeric ion channels directly activated by fructose ligands [53,60], while others may form heteromeric receptor complexes composed of co-receptors like DmGr64f and other tuning GRs [8].

With the widespread availability of molecular genetics tools such as RNA interference (RNAi) and clustered regularly interspaced short palindromic repeats (CRISPR)-mediated gene editing, alongside advanced techniques like calcium imaging assays and electrophysiological recordings, researchers have begun to uncover the molecular identities and physiological functions of GRs in various agricultural pests. For instance, in the cotton bollworm, *Helicoverpa armigera*, GRs such as HaGr6 and HaGr10 [61] act as sugar receptors, detecting D-fructose, D-galactose, and sucrose. Notably, HaGr10 is essential for sensing lower sucrose concentrations in larvae, while HaGr6 is crucial for detecting higher sucrose levels in adults [61]. In related moth and Hemiptera species, including the fall webworm, *Hyphantria cunea*, the silkworm, *Bombyx mori*, the common cutworm, *Spodoptera litura*, and the silverleaf whitefly, *Bemisia tabaci*, several sugar receptors have been identified. These include HcGr76 [62], BmGr9 [63], and SlGr8 [64] BtGr1 [65], BtGr11 [66]. Notably, the honeybee, *Apis mellifera*, possesses the smallest known GR repertoire among sequenced insects, comprising only 12 receptors [67]. Among these, AmGr1 detects sucrose and glucose, AmGr2 likely functions as a co-receptor, and AmGr3 is specialized for fructose detection [68,69].

GRs are crucial for host recognition and seeking behavior across diverse insect species. For instance, in the cotton bollworm, the receptor HaGr195 specifically detects proline, an amino acid prevalent in plant tissues, allowing the insect to locate suitable hosts [70]. The specificity of plant recognition in the cotton bollworm highlights the potential of targeting its GRs for precise pest management strategies. In mosquitoes, GRs such as AaGr2 and AaGr3 in are essential for detecting CO_2_, which is crucial for locating hosts for blood-feeding [48,71,72,73]. Targeting these receptors with genetic or chemical interventions could interfere with the mosquito’s host-seeking behavior, offering a promising avenue for controlling disease vectors. Similarly, in the fall webworm, *Hyphantria cunea*, GRs like HcGr1 and HcGr3 mediate a strong response to CO_2_, a signal often associated with plant respiration, aiding in host selection. HcGr2, however, has an inhibitory role, highlighting the complexity of interactions between GRs and CO_2_ [74]. Understanding these dynamics provides a basis for designing strategies that disrupt CO_2_-mediated host recognition, potentially reducing damage caused by this pest species.

## 3. Olfactory Receptors (ORs)

The olfactory system in insects is a highly specialized and essential sensory apparatus that plays a pivotal role in their survival, reproduction, and ecological interactions. While the gustatory system detects non-volatile chemicals, the olfactory system enables insects to sense volatile compounds, allowing them to find food, mates, and suitable habitats, as well as to avoid predators and repellents [75,76,77,78]. ORs [79], the molecular sensors of this system, are critical to these functions and have emerged as promising targets for the development of novel insecticides aimed at controlling harmful insect populations. ORs are typically composed of a divergent odorant-sensing receptor and a conserved odorant receptor co-receptor (Orco) [80,81]. Structurally, ORs are characterized by seven hydrophobic transmembrane domains (Figure 2B), a hallmark of this protein family [82,83]. Recent cryo-EM studies have resolved the structures of ORs and Orco, providing critical insights into their function [84,85]. Odor-tuning ORs assemble with Orco to form ligand-gated ion channels, and this unique combination allows insects to detect a vast range of volatile compounds in their environment.

The fruit fly, *Drosophila melanogaster*, serves as a model organism for understanding olfactory systems. It possesses 62 ORs that are highly specialized to detect diverse odorants [78,82,86,87]. Mosquitoes, including *Aedes aegypti* and *Anopheles gambiae*, which are vectors for diseases such as malaria, dengue, and Zika virus, rely heavily on their olfactory systems to locate hosts [88,89]. Specific ORs in these species are finely tuned to detect human body odors, carbon dioxide, and other host-related volatiles [71,90,91,92]. Disrupting these ORs impairs host-seeking behavior, offering a promising avenue for controlling mosquito populations [93,94]. Notably, in the southern house mosquito, *Culex quinquefasciatus*, the CqOr136 receptor is responsible for detecting DEET (N,N-Diethyl-meta-toluamide), one of the most widely used insect repellents. Genetic knockdown of CqOr136 results in a loss of aversion to DEET, highlighting its critical role in olfactory-mediated avoidance behavior [95]. Harmful agricultural pests, such as the cotton bollworm, *Helicoverpa armigera*, and the pea aphid, *Acyrthosiphon pisum*, also depend on their olfactory systems to locate crops and mates [96,97,98,99]. The ORs in these insects are highly sensitive to plant volatiles, enabling them to identify and target specific crops. For example, the cotton bollworm uses ORs to detect floral and vegetative volatiles, which guide its oviposition and feeding preferences [100,101,102]. Similarly, the pea aphid’s ORs are adapted to recognize volatile compounds from host plants, making them essential for its survival and reproduction [99]. Social insects like ants and bees exhibit advanced olfactory systems. In ants, ORs are crucial for detecting pheromones used in communication, colony coordination, and foraging [103,104]. In bees, ORs are vital for locating flowers, recognizing hive members, and maintaining social structure [105,106].

The critical role of insect ORs in host-seeking, mate-finding, and social behaviors makes them a promising target for pest control. Disrupting OR-mediated processes through genetic, chemical, or molecular approaches could impair pests’ ability to locate crops or hosts, reduce mating success, or disrupt social coordination, offering targeted pest management solutions with minimal non-target effects.

## 4. Ionotropic Glutamate Receptors (IRs)

IRs [107] represent another important class of receptors involved in taste sensation. The structural organization of IRs consists of tetrameric subunits (Figure 2C). In fruit flies, the IR family consists of 66 members, which exhibit a remarkable ability to detect a wide range of chemical stimuli, including salts [18,19,20], amino acids [10,11], amines [108], heavy metals [109,110], carbonation [111] and fatty acids [13,14]. DmIr25a and DmIr76b appear to act as co-receptors together with many other IRs and play a key role in detecting various tastants [11,18,108,111], underscoring their broad functionality in gustatory perception.

In addition to their role in taste, IRs are also involved in olfaction, working alongside odorant receptors to detect a wide range of odors [107]. Interestingly, IRs also contribute to thermosensation and hygrosensation, enabling fruit flies to respond to temperature and humidity changes. For example, the thermosensory complex involving DmIr21a and DmIr25a is essential for sensing both warm and cool temperatures, while DmIr40a and DmIr93a are necessary for humidity detection [112,113,114].

While the fruit fly has traditionally served as the primary model organism for studying ionotropic receptors, the significance of IRs extends well beyond this species, influencing a diverse array of insect behaviors. In mosquitoes, IRs are crucial for various aspects of their life cycle, particularly in host-seeking behavior and oviposition site selection [115]. Studies have demonstrated that *Anopheles aegypti* IRs, such as AaIr8a and AaIr25a, are involved in detecting human-derived odorants essential for blood feeding [90]. Moreover, in the mosquito, *Anopheles coluzzii*, AcIr76b has been implicated in sensing fatty acids, which are vital for female mosquitoes when selecting suitable oviposition sites [116]. Similarly, in the hawkmoth, *Manduca sexta*, MsIr8a detects carboxylic acids emitted from feces, such as 3-methylpentanoic acid and hexanoic acid, helping females avoid overlapping oviposition sites [117]. The tea green leafhopper, *Empoasca onukii*, utilizes the EoIr25a receptor to recognize the tea plant volatile 1-phenylethanol for locating oviposition sites [118]. In the cotton bollworm, *Helicoverpa armigera*, HaIr1.2 and HaIr75d have been associated with searching for oviposition sites, showing significantly higher expression levels in females after mating [119]. There is also a historical precedent for using sweet vinegar solutions to trap moths, and recent research has identified MsIr8a, MsIr64a, MsIr75q1, and MsIr75q2 as putative acid receptors in the oriental armyworm, *Mythimna separate* [120]. SfIr8a and SfIr75q.2 of the fall armyworm (*Spodoptera frugiperda*) exhibit responses to 8-10 carbon fatty acids and their corresponding aldehydes when expressed in *Xenopus oocytes* [121]. In the turnip moth, *Agrotis segetum*, AsIr75p.1 and AsIr75q.1 are responsive to hexanoic acid and octanoic acid, respectively [122]. Additionally, 17 IRs have been identified in the parasitoid wasp, *Microplitis mediator* [123]. Among these, MmIr64a1 responds to volatiles from host plants, whereas MmIr64a2 detects (Z)-9-tetradecenal, a critical component of the sex pheromone released by *Helicoverpa armigera*, the preferred host of *Microplitis mediator* [124]. In summary, this breadth of research highlights the diverse functions of IRs across various insect taxa, revealing their fundamental roles in behavior and ecological interactions, and underscores the potential for developing novel pest management strategies by targeting these receptors.

## 5. Other Families of Chemosensory Receptors

In addition to GRs, ORs and IRs, several other families of chemosensory receptors have been identified. These include pickpocket (PPK)/epithelial sodium channels [125,126], alkaliphile (Alka) channels [32], transient receptor potential (TRP) channels [127,128,129,130], and otopetrin (Otop) channels [21,22]. Each of these receptor families possesses distinct functional characteristics, which together create a complex framework for chemosensation in insects.

### 5.1. PPK Channels

In fruit flies, the PPK channel family (Figure 2D) includes approximately 30 members, each characterized by unique functional properties and tissue-specific expression patterns [126,131,132]. Notably, DmPPK28, found in gustatory receptor neurons in adult fruit flies, plays a crucial role in mediating taste responses to water [125,126]. DmPPK23 and the cells expressing it play an important role in the peripheral sensory system that determines courtship behavior in *Drosophila melanogaster* [131]. DmPPK25, required for normal male response to females, is expressed at the highest levels in a single sexually dimorphic gustatory neuron of most taste hairs on legs and wings, but not in neurons that detect courtship-inhibiting pheromones or food [133]. In mosquitoes with larvae that live in a water environment, another PPK member, AaPPK301, is responsible for water detection during egg laying and larval development [134].

### 5.2. Alka Channels

Insects, such as beetles [135] and fruit flies, are sensitive to alkaline pH levels in their environment. Recent research has discovered that fruit flies utilize an alkaliphile chloride channel, known as Alka, to detect noxious alkaline pH (Figure 2E). Alka is expressed in a subset of GRNs and is essential for sensing high pH levels [32]. Like olfactory sensory neurons in mice, the chloride ion concentrations inside the fruit fly’s GRNs are higher than those outside the cells. When exposed to high pH in the environment, Alka channels are activated, leading to the efflux of chloride ions. This chloride efflux causes depolarization of the GRNs, triggering the generation of action potentials. Consequently, the fruit fly senses the external alkaline pH [32]. In other insects, such as the silkworm, *Bombyx mori*, the itch mite, *Sarcoptes scabiei*, and the fall armyworm *Spodoptera frugiperda* [136], the pH-sensitive chloride channel (pHCl) related to the fly Alka channel can be irreversibly activated by insecticides ivermectin and emamectin benzoate [41,137,138]. Since both Alka and pHCl channels are pH-sensitive and belong to the same ligand-gated chloride channel (LGCC) family [32,139], their functional similarities highlight their potential as molecular targets for insecticide development.

### 5.3. TRP Channels

The TRP channel family [140] plays a crucial role in insect taste sensation (Figure 2F), with specific channels like TRPL [129] and TRPA1 [127,128,130] having distinct functions. TRPL is expressed in GRNs and is a sensor for unpalatable but non-toxic tastants, such as camphor. Notably, chronic exposure to camphor causes downregulation of TRPL protein expression, thereby reducing the insect’s taste sensitivity to camphor. This adaptive response likely helps the insect modulate its reaction to persistent, non-harmful stimuli [129]. In addition to TRPL, TRPA1 channels are also present in GRNs and are sensitive to reactive electrophiles and aristolochic acid, allowing insects to detect these specific chemical cues [128,130]. TRP channels play a critical role in enabling agricultural and sanitary pests to detect and respond to chemical cues in their environment [130,141,142]. In the brown planthopper, *Nilaparvata lugens*, NlTRPL is crucial for selecting suitable egg-laying sites [143]. In the mosquito, *Aedes aegypti*, AaTRPA1 channels are directly activated by compounds like catnip and cinnamodial; mosquitoes lacking AaTRPA1 lose their aversion to these substances [144,145]. Similarly, TRPA1 channel agonists, such as β-caryophyllene, β-citronellal, octanoic acid, and decanoic acid, deter the red imported fire ant (*Solenopsis invicta*) by activating SiTRPA1 channels [146]. In the red flour beetle, *Tribolium castaneum*, TcTRPA1 mediates repellent responses to citronellal [147]. These findings highlight TRP channel agonists as a promising chemical platform for developing novel pest control strategies.

### 5.4. Otop Channels

The Otop channel family represents a highly conserved group of proton-selective ion channels found across diverse species, including worms, insects, and mammals. First identified in mammals, Otop channels were initially linked to vestibular function, specifically otolith formation in the inner ear. Subsequent research revealed their broader role as proton-selective channels, making them essential for acid sensing across different taxa. In mammals, Otop1 is crucial for detecting sour taste, mediating responses to protons by depolarizing sour-sensitive taste cells in the tongue [148,149]. This foundational discovery highlighted the importance of Otop channels in sensory perception and paved the way for investigations into their roles in other species. In fruit flies, an Otop family member, OtopLa (Figure 2G), is both necessary and sufficient for acid detection. OtopLa enables fruit flies to respond to low concentrations of acids such as acetic and citric acid, driving their attraction to acidic food sources. These findings demonstrate that the Otop family plays a universal role in mediating proton-dependent acid sensing across insects and mammals [21,22]. This discovery is highly significant as it changes the traditional notion that insect and mammalian chemosensory receptors are fundamentally distinct and unrelated.

In addition to Otop channels, acids are also detected by ionotropic receptors (IRs) in fruit flies [23,24]. This finding suggests that acid sensation in flies may involve multiple sensory transduction pathways. Otop channels, as proton-selective ion channels, are broadly tuned to detect various acids, including citric acid, malic acid, and acetic acid, by directly responding to protons. However, the mechanism by which IRs detect acids remains to be elucidated. It is likely that IRs recognize the specific structures of carboxylic acid moieties rather than directly sensing protons [23,24]. This complementary functionality highlights the complexity of acid sensation in flies, where distinct receptor systems may operate to detect different chemical aspects of acidic stimuli. Since acids are crucial to pests’ feeding and reproductive processes, insect-specific Otop agonists or antagonists could be promising candidates for pest control.

## 6. Food Mechanosensation in Insects

Detecting food texture is crucial for insects’ survival and ecological success. Food mechanosensation allows insects to perceive physical attributes such as hardness, softness, and viscosity, which are as important as taste in guiding food preference and foraging behavior. Understanding the mechanisms behind food texture detection offers insights into insect biology and potential strategies for pest control, particularly in agriculture.

Recent advances have revealed the role of specialized mechanoreceptors in food texture detection, with the Transmembrane Channel-like (TMC) protein being a key discovery (Figure 2H) [150,151,152]. TMC is evolutionarily conserved across species, from worms to mammals, underscoring its fundamental role in mechanotransduction [150,153,154,155,156]. In mammals, mutations in TMC1 lead to deafness, highlighting its importance in hearing [151,157]. In fruit flies, TMC is essential for distinguishing between soft and hard foods. Loss of TMC impairs this ability, emphasizing its significance in food texture sensation. TMC is expressed in multidendritic neurons of the fruit fly labellum (md-L), which are specialized for food texture detection [150]. Additionally, OSCA/TMEM63 channels, another class of mechanoreceptors, are required for sensing food grittiness in these neurons [158]. Alongside md-L neurons, bipolar-type mechanosensory neurons in the fruit fly labellum also play a role in perceiving food mechanics. Mechanosensitive TRP channels, such as Nanchung [159], NOMPC [160], and Inactive [161], play key roles in this process. Together with TMC and OSCA/TMEM63, these TRP channels respond to various mechanical stimuli, illustrating the complexity of insects’ mechanosensory systems for evaluating food.

Insects’ mechanosensitive channels also hold potential as targets for insecticide development. For instance, pymetrozine abolishes sound-induced transduction currents in the desert locust, *Schistocerca gregaria* [162] and reduces action potential firing in cockroaches, *Periplaneta americana* [163]. Furthermore, the NlNan and NlIav channels, co-expressed in Xenopus laevis oocytes, are sensitive to pymetrozine in the brown planthopper, *Nilaparvata lugens* [164]. These findings highlight the potential of mechanosensitive channels as molecular targets for pest control.

## 7. Precision Pest Control Strategies

Precision pest control techniques are rapidly evolving, offering a more sustainable and targeted approach to managing both agricultural and human-harming pests. These methods are designed to minimize environmental impact while addressing the growing challenges posed by a variety of pests that affect human health, agriculture, and ecosystems.

For agricultural pests, innovative approaches include genetically modified (GM) crops that alter plant defense mechanisms, such as enhanced production of natural insecticides [165] or the manipulation of plant volatiles to deter pests [37]. Beyond agriculture, precision pest control strategies are also applied to combat human-harming pests such as mosquitoes, ticks, and flies. These pests are vectors of diseases such as malaria, lyme disease, and dengue fever, which affect millions of people globally. Genetic modification of mosquitoes has led to the development of strains that are resistant to disease transmission [38] or that cannot reproduce, thus reducing population sizes [39].

Technological advancements, including CRISPR gene editing, have allowed for more precise interventions in pest populations. For instance, gene drive systems can be used to spread genetic modifications rapidly through populations, offering a powerful tool for managing pests that are resistant to traditional control methods. Even in haplodiploid pests, the CRISPR gene drive systems can be effectively applied to control the globally invasive common wasps [166]. Additionally, biotechnological advancements have led to the development of bioinsecticides targeting specific pests by disrupting critical behaviors like feeding or reproduction. One of the most promising areas in pest control involves insect chemosensory systems, which play a central role in behaviors such as host-seeking, feeding, and mating. By disrupting the chemoreception mechanisms in pests—whether through the use of chemicals [167], GM crops [168], or pheromone traps [169]—scientists can effectively reduce pest populations without harming non-target species.

### 7.1. Genetically Modified Crops

Genetically engineered crops offer an innovative solution to pest control by manipulating the biosynthesis of secondary metabolites that either repel herbivorous insects or reduce the production of compounds that attract them. This approach has been especially valuable in reducing the need for chemical pesticides, which can have adverse environmental effects. Insect chemosensory receptors play a central role in how pests detect these plant secondary metabolites. Many insects rely on their chemoreceptor systems to locate suitable food sources and oviposition sites, and these receptors are often tuned to detect specific plant metabolites, including secondary metabolites that signal plant defense mechanisms. One example includes the modification of the thale cress, *Arabidopsis thaliana* to decrease glucosinolate levels. Many Lepidopteran pests, such as generalist *Helicoverpa armigera* and the specialist *Plutella xylostella*, which use glucosinolates in host-plant recognition [168]. Feeding by *Helicoverpa armigera* and *Plutella xylostella* larvae was 2.1 and 2.5 times less, respectively, on genetically engineered thale cress than on wild-type plants [168]. Furthermore, mutant plants lacking nonvolatile indole glucosinolates and volatile aliphatic glucosinolate breakdown products exhibited decreased oviposition attractiveness beyond that of the progenitor mutants for *Plutella xylostella* [37]. Further research has shown that two chemosensory receptors, PxOr35 and PxOr49, are indeed essential for glucosinolates to drive the host preference for the thale cress [170].

Additionally, GM plants-mediated RNAi or host-induced gene silencing (HIGS) is a promising agricultural pest control method that is highly pest-specific and has less of an impact on the environment [171]. HIGS is effective against a wide range of viruses, fungi, nematodes and insects, and HIGS products have been launched. The researchers developed transgenic tobacco and tomato crops targeting the pest chitinase gene, which significantly reduced the survival and overall growth of the cotton bollworms, *Helicoverpa armigera* [172]. Similarly, transgenic rice targeting the pest fatty acyl-CoA reductase gene through HIGS demonstrated high resistance to the rice stem borer, *Chilo suppressalis* [173]. Insect chemoreceptor genes have emerged as promising targets for HIGS due to their critical roles in pest physiological functions, including feeding, mating, egg-laying, and reproduction. For instance, knocking out the BmGr66 gene in the silkworm, *Bombyx mori*, altered their feeding preferences [174]. Similarly, silencing SlGr206 in the common cutworm, *Spodoptera litura* diminishes the larvae’s ability to forage for five crucial host odorants [175], highlighting the potential of gene-specific interventions in pest control.

These findings underscore the potential of using genetically engineered crops in combination with insect sensory receptor manipulation. By modifying crops to either enhance or suppress the production of secondary metabolites, it is possible to either attract beneficial organisms or repel pests more effectively. The interplay between plant chemistry and insect chemoreception can thus be leveraged to develop more targeted, sustainable pest management strategies that minimize environmental impacts and reduce reliance on chemical insecticides.

### 7.2. Sterile Insect Technique (SIT)

SIT has been successfully employed to control mosquito populations by suppressing reproduction. For instance, releasing sterile male *Aedes aegypti* into the wild has proven effective in reducing population sizes by preventing successful mating. One notable example involves engineering the gene β2-tubulin in *Aedes aegypti*, which can result in sterile males while leaving other physiological functions intact, enhancing the sustainability of population suppression efforts [39].

Building on these achievements, targeting specific sensory receptors critical for mating behaviors offers a promising avenue to enhance SIT. Insect sensory receptors play pivotal roles in courtship, copulation, and oviposition across diverse insect species. Recently researchers have found that linalool fumigation improves mating competitiveness of sterile male via enhance the expression of chemosensory receptors, the CpOr3a, CpOr3b, and CpOr5, in the global fruit pest *Cydia pomonella* [176]. These findings highlight the innovative role of chemosensory receptors in strengthening the effectiveness of the SIT for pest control. Furthermore, in the brown planthopper, *Nilaparvata lugens*, mutants of the receptor gene NlGr23a result in sterile males [40], whereas NlGr7 has been shown to regulate fecundity [177]. These findings indicate that the chemosensory receptors can also serve as direct targets for SIT, broadening their potential applications in pest management strategies.

By integrating these insights, insect sensory receptors could be exploited to refine SIT applications. For example, designing interventions that modify these sensory pathways in males could enhance the competitive advantage of sterile males, further reducing reproductive success in wild populations. Additionally, impairing oviposition-related chemosensory receptors in pest species could suppress egg-laying and reduce population growth. Therefore, leveraging sensory receptor-based strategies alongside sterile insect release not only enhances the precision of SIT but also opens doors to environmentally sustainable pest management, minimizing reliance on broad-spectrum insecticides.

### 7.3. Specific Insecticides Targeting Pest Sensory Receptors

Unlike mammalian chemosensory receptors, which are primarily G-protein-coupled receptors (GPCRs), insect chemosensory receptors are structurally distinct ion channels [178]. This difference is critical as it minimizes the potential for cross-reactivity with mammalian systems, ensuring that insecticides targeting these receptors are highly specific to pests, and not harmful to humans or non-target organisms.

A number of insecticides have already been developed that take advantage of insect sensory receptors. For instance, DEET has been shown to inhibit feeding behavior in *Drosophila melanogaster* by targeting Gr89a, a gustatory receptor involved in feeding regulation [26]. This highlights the potential of chemosensory receptors, particularly taste receptors, as viable targets for disrupting pest feeding behaviors. Similarly, research has demonstrated that the mechanosensitive channels, such as Nanchung and Inactive in *Drosophila melanogaster*, serve as molecular targets for two commercial insecticides, pymetrozine and pyrifluquinazon, respectively [179]. These insecticides disrupt the normal function of the mechanosensory system, making them effective in controlling pests while avoiding harmful effects on non-target species.

The use of insect sensory receptors as targets for pest control is particularly promising due to the specificity of these ion channels. Since many of these receptors are highly tuned to particular chemical cues, disrupting their function can significantly impair an insect’s ability to feed, reproduce, or locate suitable habitats [180]. This targeted approach not only ensures greater efficacy against pests but also minimizes off-target effects, making it a safer and more sustainable alternative to traditional chemical pesticides.

Despite these advances, many chemosensory and mechanosensory receptors involved in insect feeding and reproduction remain poorly understood, and their potent ligands are largely unknown. To address this gap, there is a growing need for systematic “deorphanization” efforts to identify the natural ligands for these receptors. Emerging technologies, such as cryo-EM, AlphaFold-based structural prediction, and artificial intelligence (AI)-assisted drug screening, are revolutionizing the identification of these ligands and enabling a deeper understanding of receptor structures [181].

By obtaining high-resolution 3D structures of insect sensory receptors in both their resting and ligand-bound states, researchers can unravel the molecular mechanisms behind ligand binding and receptor gating. This knowledge is essential for developing more effective and targeted insecticides. In particular, AI-driven drug screening allows for the rapid identification of potential receptor-specific agonists and antagonists, accelerating the discovery of novel insecticides [182]. This approach promises to provide insecticides with unparalleled specificity, further enhancing the sustainability and safety of pest management strategies.

As our understanding of insect chemosensory and mechanosensory receptors continues to improve, these receptors are poised to become a promising focus of insecticide development. Their structural diversity and role in crucial pest behaviors make them ideal targets for the design of next-generation insecticides, offering new avenues for pest control that are both effective and environmentally friendly.

Overall, the integration of genetically modified crops, the sterile insect technique, and insecticides targeting sensory receptors offers a multi-faceted approach to pest management that is both highly effective and environmentally friendly. These strategies, combined with advanced technological tools, hold great promise for the future of pest control, paving the way for sustainable agricultural practices and improved public health outcomes.

## 8. Perspective

While much of the existing research on insect chemosensation and mechanosensation focuses on the well-established model organism *Drosophila melanogaster*, the taste receptors of other economically significant pests, including aphids, locusts, weevils, beetles, moths, caterpillars, and whiteflies, remain largely underexplored [183,184]. These species, which are major agricultural and ecological threats, have complex and diverse feeding behaviors that are not yet fully understood at the molecular level. This gap in research is a major limitation, as it hinders the development of receptor-based pest control strategies that could target a broader range of harmful species [185]. Insects from different ecological niches and taxonomic groups may possess unique receptor profiles that reflect their specialized feeding habits and environmental adaptations. Consequently, the receptor systems of these lesser-studied pests could differ significantly from those of the widely studied models, such as *Drosophila melanogaster*. For example, aphids, known for their phloem-feeding behavior, might have distinct taste receptors compared to locusts, which are more voracious and consume a broader range of plant tissues. Similarly, caterpillars, with their highly specialized larval feeding habits, may feature unique receptor adaptations critical to their survival and development [186].

Although modern molecular biology techniques, such as CRISPR-mediated gene editing and transgenes, have been successfully applied to non-model organisms, the genetic complexity of many agricultural pests presents unique challenges. Unlike the model organism *Drosophila melanogaster*, agricultural pests often possess larger chromosome numbers, more intricate genetic inheritance, and distinct feeding behaviors, which complicate the application of these advanced techniques [187]. Expanding the study of sensory receptors in these economically important pests should therefore be a key area of future research. Such an effort could reveal novel targets for pest control interventions that are tailored to the specific sensory mechanisms of each species [188]. Furthermore, such research may uncover critical differences in receptor sensitivity, ligand-binding patterns, and neural processing between different insect groups, facilitating the development of more species-specific and environmentally sustainable pest management strategies.

Additionally, translating laboratory findings into real-world applications remains a significant challenge. The behavior of insects in natural environments is influenced by a multitude of factors, including habitat conditions, seasonal variations, and interactions with other species, all of which can affect the efficacy of receptor-targeting insecticides [189]. Therefore, while targeting specific sensory receptors offers considerable promise for pest control, the practical effectiveness of these strategies must be validated through rigorous field trials. Such trials are essential to determine how these interventions perform under natural conditions, to assess potential behavioral adaptations of target pests, and to minimize ecological impacts, including non-target effects on beneficial organisms.
